# Comprehensive analysis of the critical transcript function of the *DAZAP2* gene in porcine testis

**DOI:** 10.5194/aab-69-57-2026

**Published:** 2026-01-27

**Authors:** Xia Zhang, Hailong Huo, Honglin Li, Lan Zhai, Jinlong Huo

**Affiliations:** 1 Department of Biological and Food Engineering, Lyuliang University, Lyuliang, Shanxi, 033001, China; 2 College of Animal Science and Technology, Yunnan Agricultural University, Kunming, Yunnan, 650201, China; 3 Yunnan Open University, Kunming, Yunnan, 650101, China

## Abstract

The *DAZAP2* (Deleted in Azoospermia-associated Protein 2) gene encodes an azoospermia-related protein that plays key roles in spermatogenesis, cell cycle regulation, and transcriptional regulation. Here, we employed transcriptome sequencing to analyze porcine testis tissues using long-read and short-read sequencing and identified the *DAZAP2* transcripts via RT-PCR. Protein interaction analysis, GO and KEGG enrichment, and competing endogenous RNA (ceRNA) regulatory network construction were performed to elucidate its functional pathways. Furthermore, we assessed the multi-tissue expression of *DAZAP2* and the subcellular localization of the DAZAP2 protein. We identified two spliceosomes of the *DAZAP2* gene in Banna mini-pig inbred line (BMI) testicular tissue, namely DAZAP2_X1 and DAZAP2_X2, with DAZAP2_X2 being the predominant transcript. Functional enrichment analysis revealed that DAZAP2_X2 was associated with ubiquitin–protein ligase binding, positive regulation of protein monoubiquitination and Wnt signaling pathway, indicating its involvement in spermatogenesis. Additionally, we identified nine microRNAs (miRNAs) interacting with DAZAP2_X2, including ssc-miR-490-3p, ssc-miR-150, ssc-miR-107, ssc-miR-193a-3p, ssc-miR-497, ssc-miR-192, ssc-miR-383, ssc-miR-129a-5p, and ssc-miR-181a, most of which were associated with spermatogenesis. We found DAZAP2_X2 was highly expressed in the testis and bulbourethral glands and was mainly localized in the cytoplasm. These findings suggest that DAZAP2_X2 played a significant role in spermatogenesis and provide a reference for further research on spermatogenesis-related genes and regulatory pathways.

## 1 Introduction

Germ cells are foundational to sexual reproduction in organisms, playing critical roles in offspring generation and genetic diversity. Primordial germ cells, the initial germ cell population, form during development and differentiate into spermatogonia and oocytes. In mammals, germ cells serve as the foundational units for the generation of new individuals (Saitou and Yamaji, 2012). Male germ cell development involves a series of distinct processes, including meiosis, genetic recombination, haploid gene expression, acrosome, and flagellum formation, as well as chromatin remodeling and condensation. Spermatogenesis is a complex process encompassing mitotic division, meiosis, and the differentiation of spermatogenic cells (Diao et al., 2019). DAZAP2 (Deleted in Azoospermia-associated Protein 2) was initially identified in a yeast two-hybrid screen as interacting partners of the male sterility factor DAZ (Deleted in Azoospermia) (Tsui et al., 2000). The *DAZAP2* gene encodes a highly conserved 17 kDa protein that interacts with *DAZ* via DAZ-like repeats and binds to key regulatory molecules such as SARA (Smad anchor for receptor activation), the eukaryotic translation initiation factor 4G, and E3 ubiquitin ligase. These interactions regulate the stability of *DAZAP2* and its function in modulating splicing factors within nuclear speckles (Fu et al., 2015). DAZAP2 participates in various biological and pathological processes, including spermatogenesis, cell signaling, transcriptional regulation, stress granule formation during translational arrest, RNA splicing, and the pathogenesis of multiple myeloma (Behera et al., 2023). Functional studies indicate that the knockout of *DAZAP2* not only diminishes Wnt signaling activity, evidenced by reduced Tcf/ β -catenin reporter expression, but also disrupts the expression of downstream Wnt target genes (Lukas et al., 2009). Chromatin immunoprecipitation assays reveal that *DAZAP2* modulates TCF-4 affinity for DNA recognition motifs. Additionally, *DAZAP2* functions as a novel downstream target of the P38/MAPK/CREB signaling pathway, regulating various transcription factors. Promoter methylation in multiple myeloma cells leads to its epigenetic silencing, further implicating *DAZAP2* in disease pathogenesis (Li et al., 2019).

The Banna mini-pig inbred line(BMI) line exhibits a clear genetic background, and its physiological characteristics are closely similar to those of humans, making it a valuable animal model (Huo et al., 2022; Liu et al., 2023; Wang et al., 2023). The objective of this study was to investigate the molecular regulation of *DAZAP2* gene expression during spermatogenesis using transcriptome sequencing and to validate its cellular localization. Our findings lay the foundational insights into the regulatory mechanism underlying testicular spermatogenesis in the BMI and provide a theoretical basis for boar breeding.

## 2 Materials and methods

### 2.1 Samples and transcriptome sequencing

Testicular samples were obtained from four 12-month-old BMI males. We conducted a transcriptomic analysis of *DAZAP2* in BMI testes using an integrated approach combining short-read RNA-seq (Novogene Technology Co., Ltd., China) and long-read iso-seq (GrandOmics Co., Ltd., China). *DAZAP2* transcripts were visualized using the Sashimi plot function in IGV, and gene expression levels and transcript abundance were analyzed using standard computational methods (Zhang et al., 2024).

### 2.2 Sequence identification of different transcripts

The complete coding region mRNA sequence of *DAZAP2* was retrieved from the NCBI database and used as a template for primer design with Oligo7 software. Primers, labeled F1R1 (F1: ACACGGAAGTGACTACGAAC; R1: ACCAATCAGAATAATTTGGCAAC) were used for gene cloning. The PCR reaction mixture comprised 25 µ L of 2 × Premix ExTaq HS, 1 µ L each of forward and reverse primers, 5 µ L of template cDNA, and RNase-free water to a final volume of 50 µ L. PCR was performed under standard conditions with an annealing temperature of 55 °C. Afterward, we cloned the *DAZAP2* gene from the testicular tissue of BMIs.

### 2.3 Characterization of key transcripts of *DAZAP2*

The nucleotide and protein sequences of key *DAZAP2* transcripts obtained from sequencing were refined using the Lasergene 7.1 software, enabling the identification of the complete coding sequence. We conducted sequence alignment, subcellular localization, and secondary and tertiary structure predictions using Seqman, PSORT II Prediction, SPOMA, and AlphaFold3, respectively. Additionally, we utilized TMHMM-2.0, ExPASy, and SMART to predict protein physicochemical properties, hydrophilicity, and protein functional modification sites. Conserved domains and evolutionary relationships were assessed using BLAST and MegAlign.

### 2.4 Interacting proteins and regulatory network

We constructed a protein–protein interaction (PPI) network and performed GO and KEGG functional enrichment analysis on the identified proteins, with a significance threshold of P<0.05 . The identified proteins were correlated with the obtained gene expression levels. We used the annotations of the UniProt database to gain insight into biological processes associated with *DAZAP2*. Based on the competing endogenous RNA (ceRNA) regulatory mechanism, which plays an important role in post-transcriptional regulation by mediating RNA–RNA interactions, a DAZAP2-associated ceRNA network was constructed to identify potential microRNAs (miRNAs) and long non-coding RNAs (lncRNAs) involved in the regulation of DAZAP2. The ceRNA regulatory network was visualized using Cytoscape version 3.9.1.

### 2.5 Detection of expression in different tissues

The cDNA templates from BMI tissues, including the heart, liver, spleen, lungs, kidneys, and gonads, as well as accessory glands, were used to assess *DAZAP2* expression levels across various tissues. Based on the nucleotide sequences of the significant transcripts of the *DAZAP2* gene, qPCR primers (F2: ACCATGAACAGCAAAGGTCAA; R2: CTCGGACGATAGAGCTCTGAA) were designed at the exon–exon junction using Seqman and Oligo7 software. Among the candidate internal reference genes, *GAPDH* was selected as the internal control based on its relatively stable expression across samples, enabling accurate normalization of gene expression levels (Klein et al., 2011; Zeng et al., 2014). The housekeeping gene *GAPDH* (F3: CCTTCATTGACCTCCACTACATGGT; R3: CCACAACATACGTAGCACCAGCATC) served as an internal control. The qPCR results were quantified using the threshold cycle values and analyzed via the 2^−ΔΔCt^ method for relative quantification (Rao et al., 2013). For each tissue, biological replicates were included in the experimental design.

### 2.6 Subcellular localization

Transfection was performed when cell confluence reached 70 %–80 % in each well of the culture plate. Subsequently, green fluorescent protein expression was observed under an inverted fluorescence microscope to confirm successful transfection. Swine testis (ST) cells were selected for subcellular localization analysis of DAZAP2 due to their species- and tissue-specific origin, stable growth, and suitability for efficient in vitro transfection and expression. Mitochondria and nuclei in ST cells were stained with Mito Tracker and Hoechst 33342, respectively, to determine the intracellular localization of the DAZAP2 protein.

## 3 Results

### 3.1 Different alternative splicing isoforms of DAZAP2

**Figure 1:**
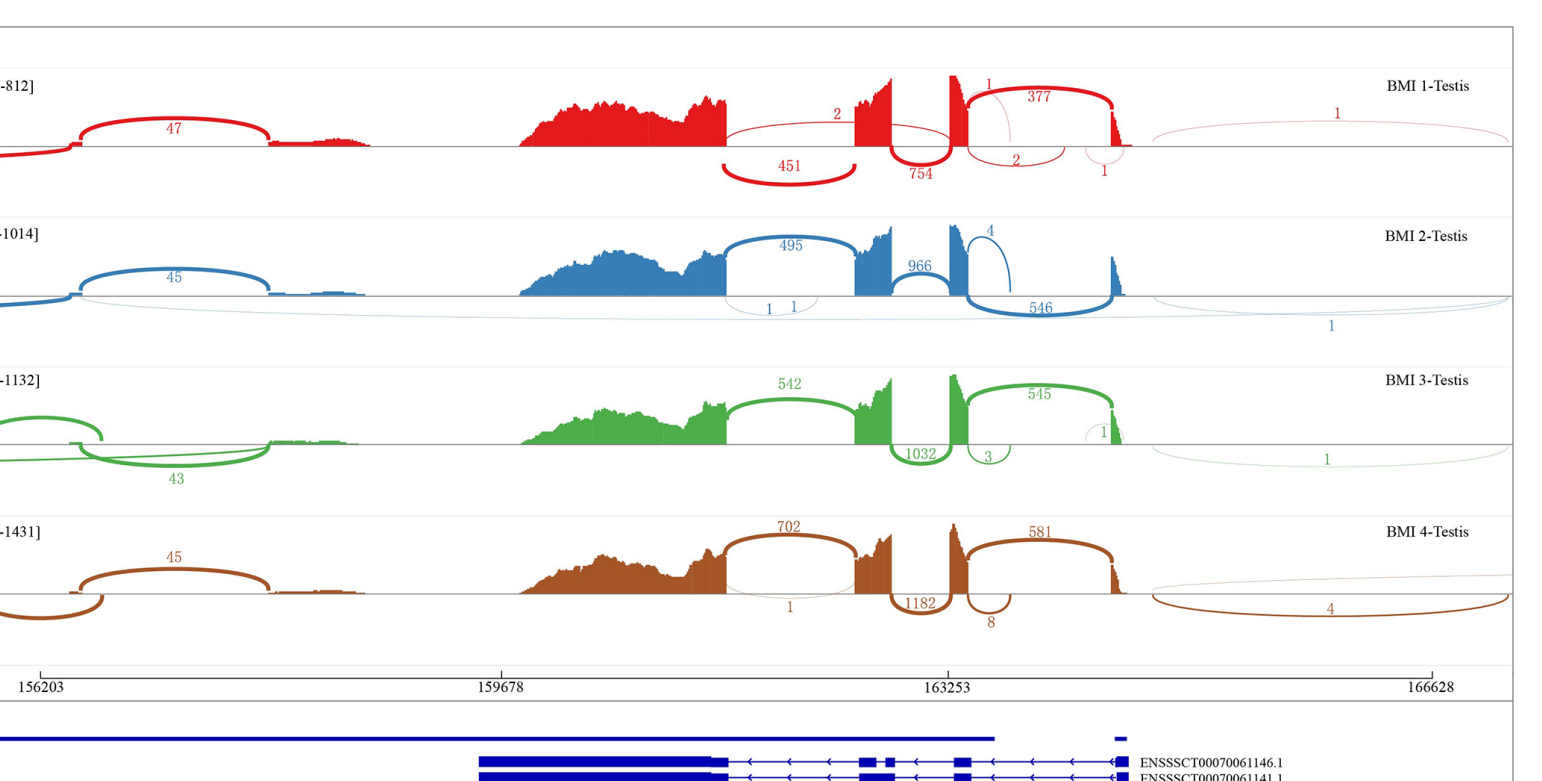
Gene structure and expression of alternative spliceosomes of porcine *DAZAP2*.

The short-read RNA-seq and long-read iso-seq results identified two alternative splicing (AS) isoforms in the BMI testes, denoted as DAZAP2_X1 (ENSSSCT00070061146.1) and DAZAP2_X2 (ENSSSCT00070061141.1) (Fig. 1). The DAZAP2_X1 isoform comprises 5 exons, with a CDS length of 441 bp (146 amino acids) and nucleotide composition of 43.99 % A + T and 56.01 % G + C. The DAZAP2_X2 isoform consists of 4 exons, with a CDS of 507 bp (168 amino acids) and nucleotide composition of 43.98 % A + T and 56.02 % G + C. In our four biological replicates, the transcript DAZAP2_X2, which encoded the longest CDS, was consistently detected, indicating its predominant role (Fig. 1).

### 3.2 Sequence detection of different transcripts of DAZAP2

**Figure 2:**
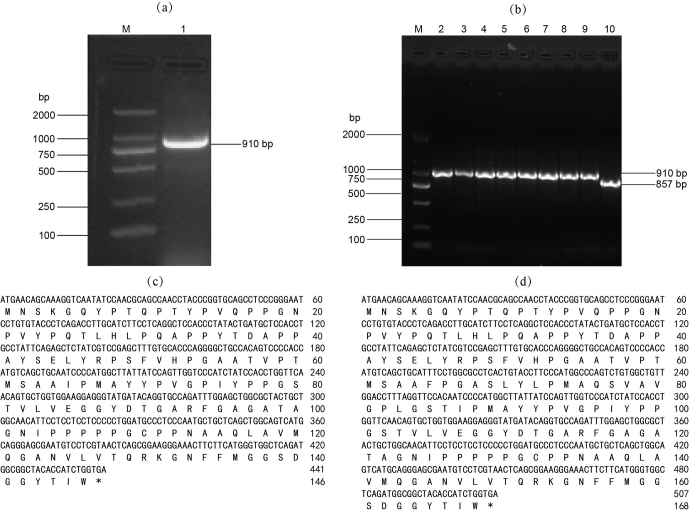
CDS and amino acid sequence of BMI *DAZAP2* alternative spliceosomes. **(a)** DAZAP2_X1; **(b)** DAZAP2_X1 and DAZAP2_X2; **(c)** sequence of DAZAP2_X1; **(d)** sequence of DAZAP2_X2.

cDNAs from BMI testicular tissue were used as a template to amplify the *DAZAP2* gene sequences. 1 % agarose gel electrophoresis confirmed the presence of 910 bp amplicon, matching the expected size (Fig. 2a). Afterwards, PCR analysis of bacterial colonies harboring pMD18-T-*DAZAP2* revealed two different fragments, suggesting the presence of alternative splicing isoforms of the *DAZAP2* gene in pig testis tissue (Fig. 2b). The nucleotide and amino acid sequences of DAZAP2_X1 isoform were shown in Fig. 2c, and those of the DAZAP2_X2 isoform were shown in Fig. 2d. Consistent with the transcriptome data, DAZAP2_X2 was identified as the predominant transcript.

### 3.3 Sequence characterization of DAZAP2_X2

**Figure 3:**
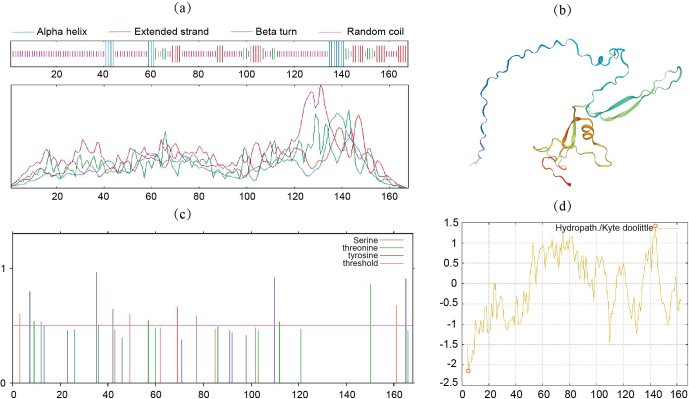
Structure of DAZAP2_X2 protein. **(a)** Secondary structure; **(b)** tertiary structure; **(c)** phosphorylation site; **(d)** hydrophobicity.

DAZAP2 protein had a molecular weight of 17.3 kDa, with the molecular formula C_792_H_1183_N_199_O_227_S_7_ and an isoelectric point of 6.69. In the secondary structure of DAZAP2_X2, α -helix accounts for 8.33 % (14 amino acids), random coil for 69.05 % (116 amino acids), extended chain for 14.88 % (25 amino acids), and β -turn for 7.74 % (13 amino acids) (Fig. 3a). The tertiary structure was consistent with the secondary structure (Fig. 3b). The DAZAP2 protein also included phosphorylation sites such as serine, threonine, and tyrosine (Fig. 3c). The N-terminus of DAZAP2 protein was hydrophobic, while the C-terminus was hydrophilic. The maximum hydrophobicity value of 1.411 was observed at the 144th amino acid, and the minimum value of - 2.133 was found at the 5th amino acid (Fig. 3d).

### 3.4 Conservation and evolutionary relationship of DAZAP2_X2 in multiple species

**Figure 4:**
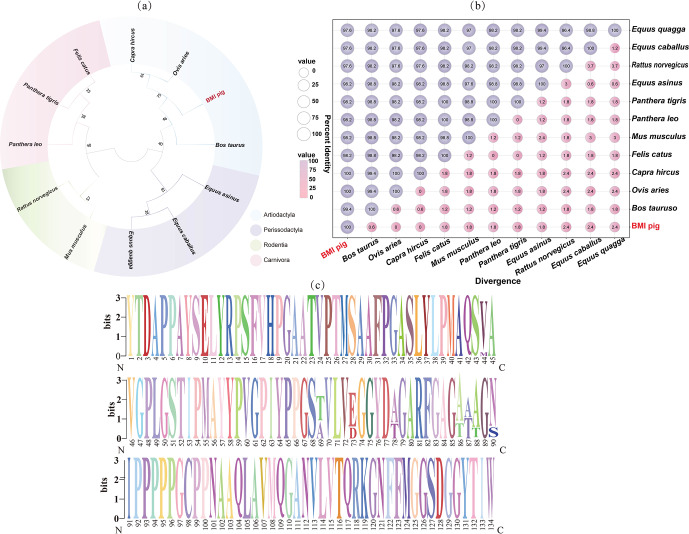
Amino acid sequence phylogenetic tree, similarity, and conserved domain analysis of BMIs and *DAZAP2* from other mammals. **(a)** Phylogenetic tree; **(b)** similarity; **(c)** conserved domain.

A comparison of DAZAP2 amino acid sequences across 12 mammalian species reveals that the DAZAP2 sequences in BMIs are highly similar (over 90 %) to those in cattle (*Bos taurus* NP_001029873), goats (*Capra hircus* XP_017903242), sheep *(Ovis aries* XP_012029646), horses (*Equus caballus* XP_003365282), zebras (*Equus quagga* XP_046515063), donkeys (*Equus asinus* XP_014702907), cats (*Felis catus* XP_003988752), tigers (*Panthera tigris* XP_007073096), lions (*Panthera leo* XP_042802501), rats (*Rattus norvegicus* XP_038934862), and mice (*Mus musculus* NP_036003) (Fig. 4a). The phylogenetic tree based on amino acid sequences of 12 species shows that donkeys, horses, and spotted sheep are clustered in one branch; cats, tigers, and lions are in another branch; and rats and mice are in a third branch – in addition, horses, pigs, cattle, sheep, and goats are clustered together, indicating a close evolutionary relationship among these species (Fig. 4b). A Weblogo diagram of the conserved domains of DAZAP2 in the 12 species further indicates that DAZAP2 is highly conserved across species (Fig. 4c).

### 3.5 Protein–protein interactions of DAZAP2_X2

**Figure 5:**
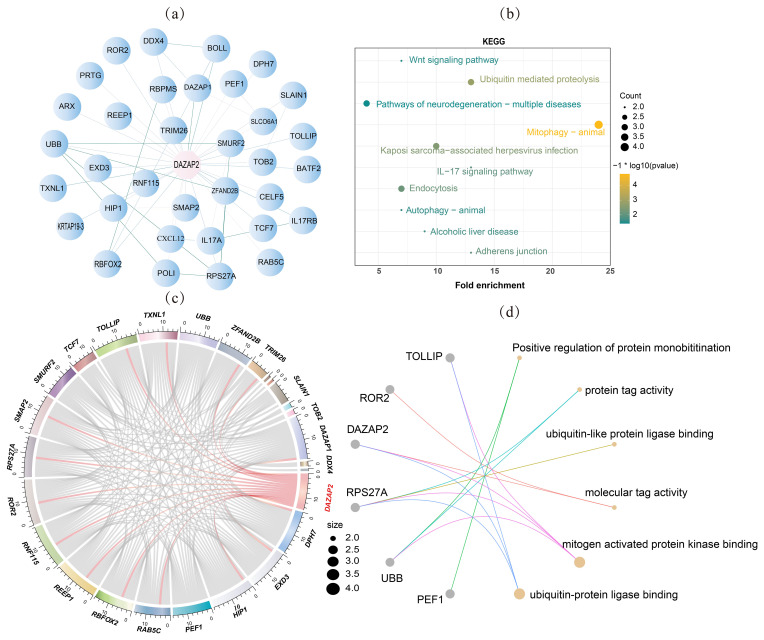
DAZAP2 protein interaction relationship. **(a)** Interacting protein network; **(b)** KEGG enrichment analysis diagram; **(c)** correlation analysis chord diagram; **(d)** GO enrichment analysis diagram.

The construction of the protein network revealed porcine DAZAP2 interacted with multiple proteins, including tripartite motif containing 26 (TRIM26); DAZ-associated protein 1 (DAZAP1); boule homolog, RNA-binding protein (BOLL); DEAD-box helicase 4 (DDX4); Toll-interacting protein (TOLLIP); RNA-binding fox-1 homolog 2 (RBFOX2); SMAD-specific E3 ubiquitin protein ligase 2 (SMURF2); zinc finger AN1-type containing 2B (ZFAND2B); ribosomal protein S27a (RPS27A); ubiquitin B (UBB); DNA polymerase iota (DNA polymerase iota, POLI); keratin-associated protein 19-3 (KRTAP19-3); and RNA-binding protein, mRNA processing factor (RBPMS) (Fig. 5a). These proteins were mainly involved in protein synthesis, chromatin structure maintenance, gene expression regulation, stress response, and germ cell development, all of which played important roles in spermatogenesis.

KEGG pathway enrichment analysis of DAZAP2-associated proteins identified 10 pathways with P<0.05 . These proteins were primarily enriched in the Wnt signaling pathway, ubiquitin-mediated proteolysis, neurodegeneration pathway, multiple diseases, mitochondrial autophagy, Kaposi's sarcoma-associated herpesvirus infection, IL-17 signaling, endocytosis, autophagy, alcoholic liver disease, and adherens junction (Fig. 5b). Gene ontology (GO) enrichment analysis revealed that these proteins were mainly involved in processes such as positive regulation of protein monoubiquitination, protein tag activity, ubiquitin-like protein ligase binding, mitogen-activated protein kinase binding, and ubiquitin–protein ligase binding (Fig. 5c). Correlation analysis of transcriptome data revealed that *DAZAP2* expression was significantly correlated with the expression of *DPH7*, *EXD3*, *HIP1*, *RAB5C*, *RBFOX2*, *REEP1*, *RNF115*, *ROR2*, *RPS27A*, *SMAP2*, *TOLLIP*, *TXNL1*, *ZFAND2B*, *TRIM26*, and *DDX4* ( P<0.05 ) (Fig. 5d).

The lines (edges) typically represent the interactions between proteins, and the thickness of the lines usually indicates the strength of these interactions.

### 3.6 Construction of ceRNA regulatory network of DAZAP2_ X2

**Figure 6:**
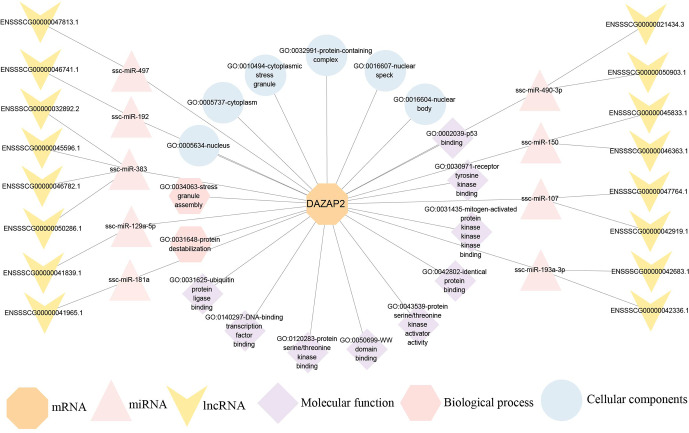
Functional annotation and ceRNA regulatory network of *DAZAP2*.

Functional annotation of *DAZAP2* identified 17 gene ontology (GO) terms across cellular components, molecular functions, and biological processes. Cellular components included nuclear bodies, nuclear speckles, protein domain complexes, cytoplasmic stress granules, cytoplasm, and nucleus. Molecular functions encompassed binding to ubiquitin protein ligases, DNA-binding transcription factor, protein serine/threonine kinase, WW domain, mitogen-activated protein kinase, receptor tyrosine kinase, and p53. Biological processes mainly involved protein instability and stress granule assembly. ceRNA regulatory network analysis revealed that *DAZAP2* expression in BMIs was mainly regulated by nine miRNA targets, including ssc-miR-490-3p, ssc-miR-150, ssc-miR-107, ssc-miR-193a-3p, ssc-miR-497, ssc-miR-192, ssc-miR-383, ssc-miR-129a-5p, and ssc-miR-181a. Additionally, 16 lncRNAs were found to compete with these miRNAs for binding to *DAZAP2* (Fig. 6).

### 3.7 Multi-tissue expression results

**Figure 7:**
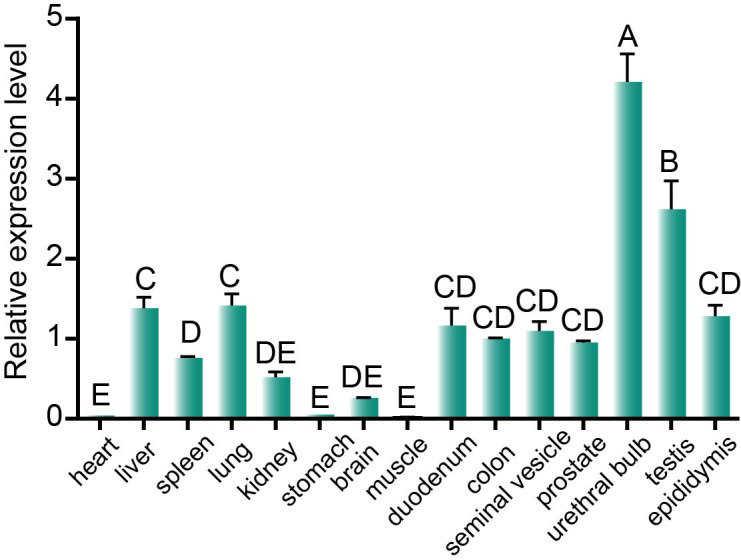
Multi-tissue expression profile of the BMI *DAZAP2* gene.

In order to investigate the expression of the *DAZAP2* gene in various tissues, qPCR was performed using *GAPDH* as the internal reference and the 2^−ΔΔCt^ method for relative quantification. The results demonstrated varying levels of *DAZAP2* expression across the 15 tissues tested. Notably, higher expression was observed in the gonadal organs, such as the bulbourethral gland, testis, and epididymis, and moderate expression was observed in the duodenum, colon, liver, lung, and prostate, while other tissues exhibited relatively low expression (Fig. 7).

Different capital letters on the bars indicate significant differences, P<0.01 . Error bars are expressed as standard deviations (SDs).

### 3.8 Subcellular localization results

**Figure 8:**
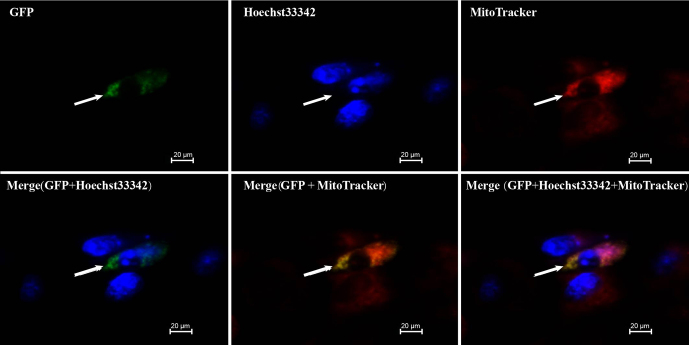
Subcellular localization of DAZAP2 protein.

Swine testis (ST) cells were transiently transfected with pEGFP-C1-*DAZAP2* and control pEGFP-C1 plasmids. Green fluorescence was observed 24 h post-transfection. Cells were then stained with MitoTracker Red CMXRos (red, mitochondrial marker) and Hoechst33342 (blue, nuclear marker) and examined using an inverted fluorescence microscope. We superimposed green, red, and blue fluorescence in the same field of view, confirming that *DAZAP2* was mainly localized in the cytoplasm (Fig. 8).

## 4 Discussion

Alternative splicing (AS) is a regulatory process in which exons of a gene's primary transcript (pre-mRNA) are spliced in various combinations, resulting in the production of mRNA isoforms and corresponding protein variants with distinct structures and functions (Blencowe, 2006). Alternative transcription initiation, splicing, and polyadenylation of pre-mRNA are pivotal mechanisms driving the extensive proteome diversity observed in eukaryotic organisms (Griffith et al., 2010). Co-transcriptional and post-transcriptional regulation enables a single gene to generate diverse mRNA isoforms (Nilsen and Graveley, 2010). In this study, PCR and molecular cloning were used to isolate the coding region sequences of two *DAZAP2* splice isoforms, DAZAP2_X1 and DAZAP2_X2. A comparison of the porcine DAZAP2 amino acid sequence with that of 11 other species revealed 100 % sequence similarity with sheep and goats and over 97 % similarity with mammals such as horses, donkeys, zebras, rats, and mice. These findings indicated that DAZAP2 was evolutionarily conserved, maintaining a stable structure, and supporting its potential for further functional analysis.

The interactions between proteins play pivotal roles in the regulation pathway of normal life activities. In this study, the protein interaction network revealed that DAZAP2 interacted with several key proteins, including TRIM26, DAZAP1, BOLL, DDX4, RBFOX2, SMURF2, ZFAND2B, RPS27A, and RBPMS. Tripartite motif containing protein 26 (TRIM26), a member of the TRIM protein family, plays a pivotal role in bladder cancer, where its knockdown suppresses cell proliferation, migration, and invasion by inhibiting the Akt/GSK3 β / β -catenin signaling pathway, highlighting its potential as a therapeutic target (Xie et al., 2021). DAZ-associated protein 1 (DAZAP1), a DAZ-interacting protein, is predominantly expressed in the testes and is essential for the reproductive system. In *DAZAP1*-deficient mice, perinatal lethality and impaired spermatogenesis are observed (Tsui et al., 2000). DAZAP2 exhibited the strongest interaction with DAZAP1, highlighting a potentially critical functional relationship between these two proteins. DAZAP1, as a high-degree hub protein within the network, likely serves as a central regulatory node, and the strong association with DAZAP2 suggests that the latter may rely on DAZAP1 to execute its biological functions. *BOLL*, a testis-specific gene, is essential for male spermatogenesis. Loss of *BOLL* expression results in spermatogenic arrest and impaired sperm maturation across various species, with defective acrosomal formation and spermatogenesis in BOLL-deficient boars (Nosková et al., 2020). DEAD-box helicase 4 (DDX4), a germ-cell-specific marker, is vital for primordial germ cell formation (Azizi et al., 2021) and spermatogenesis, and decreased *DDX4* expression is linked to male infertility (Li et al., 2010). *RBFOX2*, a member of the mammalian BBFOX family, regulates tissue-specific alternative splicing, mRNA stability (Chen et al., 2017), and translation, and it modulates TEAD1-splicing isoforms via the Hippo signaling pathway (Mitchell and Parker, 2014). SMURF1, a member of the HECT family of E3 ubiquitin ligases, causes the activation receptor and proteomic degradation of SMAD2 and SMAD3, thereby down-regulating activin TGF β signaling (Itman et al., 2011). AN1 zinc finger protein 2B (ZFAND2B) plays a critical role in maintaining protein homeostasis by facilitating the protein translocation to the endoplasmic reticulum and regulating its degradation via the proteasome. Dysregulated expression of RPS27A in sperm is associated with lower pregnancy rates (Montjean et al., 2012). RBPMS2 inhibits testicular factors and promotes oocyte development through Gator2-mediated mechanisms in zebrafish oogenesis (Wilson et al., 2024). These protein interactions provide a foundation for further investigation into the functional role of *DAZAP2*.

Competing endogenous RNAs (ceRNAs) represent a novel mechanism of gene expression regulation, where long non-coding RNAs (lncRNAs) sequester microRNAs (miRNAs), thereby disrupting miRNA binding to the 3^′^ UTR of target mRNAs and influencing downstream gene expression and cellular functions (Cai et al., 2009). lncRNAs function as ceRNAs, modulating protein expression levels by competing for miRNAs, thus playing a crucial role in regulating cellular processes (Poliseno et al., 2010). This study performed functional annotation of the *DAZAP2* gene and explored the potential ceRNA regulatory network, identifying nine miRNAs that target *DAZAP2*. ssc-miR-490-3p promotes liver cancer cell growth, migration, and metastasis by upregulating ERGIC3, facilitating epithelial–mesenchymal transition (EMT), and further enhancing cell invasion capabilities (Zhang et al., 2013). Thus, inhibition of miR-490-3p represents a promising molecular therapeutic strategy for the treatment of liver cancer. miR-150 is primarily expressed in Leydig cells, where it negatively regulates STAR expression, playing a crucial role in steroidogenesis and spermatogenesis (Niu et al., 2011). miR-107 is down-regulated in colorectal cancer tissue cell lines, with low expression correlating with poor survival in colorectal cancer patients (Fu et al., 2019). miR-193a-3p serves as a potential biomarker for the diagnosis of Alzheimer's disease, preventing neurotoxicity by targeting PTEN (Zhang et al., 2013), and also inhibits growth and invasion in cancer cells such as gastric and lung cancer (Chou et al., 2018). miR-497 is involved in fat deposition in pigs, while miR-192, implicated in various cancers, inhibits cell proliferation in MCF7 and MDA-MB-231 cells, along with cell cycle arrest in breast cancer cell lines (Chen et al., 2019). Abnormal expression of miR-383 has been linked to male infertility and testicular germ cell tumors (Tian et al., 2013). miR-129-5p enhances cell proliferation, migration, and invasion in retinoblastoma cell lines Y79 and WERI-Rb-1 by directly targeting the 3^′^ untranslated region of PAX6, activating the PI3K/AKT signaling pathway (Liu et al., 2019). miR-129-5p and miR-130a-3p regulate growth factor receptor expression during neuronal development (Glaesel et al., 2020). Additionally, miR-181a regulates spermatogonia proliferation by targeting S6K1, and its overexpression leads to spermatocyte growth arrest (Wang et al., 2021). These findings strongly suggest that the miRNAs predicted through our ceRNA network may participate in porcine spermatogenesis by regulating DAZAP2 expression. This provides robust functional evidence supporting our predictions and identifies high-priority candidate molecules for subsequent functional validation experiments.

*DAZAP2* is ubiquitously expressed across various tissues in both mice and humans, including the stomach, thyroid, spinal cord, lymph nodes, trachea, adrenal glands, and bone marrow (Li et al., 2019; Rong et al., 2007). In this study, *DAZAP2* expression was detected in various porcine tissues, with the highest expression observed in the testis and bulbourethral gland. A previous study, which utilized a DAZAP2-GFP fusion expression vector transfected into COS7 cells, demonstrated that DAZAP2 protein was primarily localized in the cytoplasm with a distinct, discontinuous distribution pattern (Shi et al., 2004). Consistent with previous findings, our study also indicated that DAZAP2 predominantly localized to cytoplasm in ST cells. Overall, the results highlighted the importance of *DAZAP2* in various biological processes, particularly in spermatogenesis, providing a theoretical foundation for further investigation into its functional role in pigs. Future studies should prioritize the functional characterization of the major DAZAP2_X2 transcript. Based on our findings, we plan to investigate the effects of DAZAP2 loss of function in reproductive cells by knocking down or knocking out DAZAP2 in pig spermatogonial stem cells or appropriate model organisms. This approach will allow direct assessment of its impact on key spermatogenic events, including germ cell proliferation, apoptosis, meiotic progression, and spermatogenesis. Our protein interaction and KEGG analyses strongly suggest that DAZAP2 is involved in the Wnt signaling pathway and ubiquitin-mediated proteolysis. We can examine the effects of DAZAP2 depletion on Tcf/ β -catenin reporter activity and the expression of downstream Wnt target genes (e.g, *AXIN2*, *CYCLIND1*). In addition, its role in regulating the stability of key spermatogenic proteins through ubiquitination can be further explored.

## 5 Conclusions

In this study, we integrated short-read and long-read sequencing techniques to reveal transcriptional regulation characteristics of *DAZAP2* in pigs. Notably, we identified two distinct *DAZAP2* transcripts, with DAZAP2_X2 being the predominant form, consisting of four exons and displaying a high conservation across 12 species. Proteins interacting with DAZAP2 were primarily involved in protein synthesis, chromatin structure maintenance, gene expression regulation, stress response, and germ cell development. Functional annotation revealed 17 proteins associated with GO terms, including nine molecular functions, two biological processes, and six cellular components. Nine miRNAs were identified as key regulators of *DAZAP2* expression. Specifically, *DAZAP2* expression was most prominent in the urethral bulb glands and testes, with its protein predominantly localized in the cytoplasm of ST cells. These findings deepen our understanding of the transcriptional regulation of spermatogenesis, laying a foundation for further investigation into its role in sperm capacitation and the acrosome reaction in pigs.

## Data Availability

The datasets presented in this study are available from the corresponding author upon request.

## References

[bib1.bib1] Azizi H, NiaziTabar A, Mohammadi A, Skutella T (2021). Characterization of DDX4 gene expression in human cases with non-obstructive azoospermia and in sterile and fertile mice. Journal of Reproduction and Infertility.

[bib1.bib2] Behera C, Kaushik R, Bharti DR, Nayak B, Bhardwaj DN, Pradhan D, Singh H (2023). PsychArray-based genome wide association study of suicidal deaths in India. Brain Sciences.

[bib1.bib3] Blencowe BJ (2006). Alternative splicing: new insights from global analyses. Cell.

[bib1.bib4] Cai Y, Yu X, Hu S, Yu J (2009). A brief review on the mechanisms of miRNA regulation. Genomics, Proteomics and Bioinformatics.

[bib1.bib5] Chen J, Cai T, Zheng C, Lin X, Wang G, Liao S, Wang X, Gan H, Zhang D, Hu X, Wang S (2017). MicroRNA-202 maintains spermatogonial stem cells by inhibiting cell cycle regulators and RNA binding proteins. Nucleic Acids Research.

[bib1.bib6] Chen P, Feng Y, Zhang H, Shi X, Li B, Ju W, Yu X, Zhang N, Luo X (2019). MicroRNA-192 inhibits cell proliferation and induces apoptosis in human breast cancer by targeting caveolin 1. Oncology Reports.

[bib1.bib7] Chou NH, Lo YH, Wang KC, Kang CH, Tsai CY, Tsai KW (2018). MiR-193a-5p and-3p play a distinct role in gastric cancer: miR-193a-3p suppresses gastric cancer cell growth by targeting ETS1 and CCND1. Anticancer Research.

[bib1.bib8] Diao S, Huang S, Chen Z, Teng J, Ma Y, Yuan X, Chen Z, Zhang H, Li J, Zhang Z (2019). Genome-wide signatures of selection detection in three South China indigenous pigs. Genes.

[bib1.bib9] Fu XF, Cheng SF, Wang LQ, Yin S, De Felici M, Shen W (2015). DAZ family proteins, key players for germ cell development. Int J Biol Sci.

[bib1.bib10] Fu Y, Lin L, Xia L (2019). MiR-107 function as a tumor suppressor gene in colorectal cancer by targeting transferrin receptor 1. Cell Mol Biol Lett.

[bib1.bib11] Glaesel K, May C, Marcus K, Matschke V, Theiss C, Theis V (2020). miR-129-5p and miR-130a-3p regulate VEGFR-2 expression in sensory and motor neurons during development. Int J Mol Sci.

[bib1.bib12] Griffith M, Griffith OL, Mwenifumbo J, Goya R, Morrissy AS, Morin RD, Corbett R, Tang MJ, Hou YC, Pugh TJ, Robertson G (2010). Alternative expression analysis by RNA sequencing. Nature Methods.

[bib1.bib13] Huo JL, Zhang LQ, Zhang X, Wu XW, Ye XH, Sun YH, Cheng WM, Yang K, Pan WR, Zeng YZ (2022). Genome – wide single nucleotide polymorphism array and whole – genome sequencing reveal the inbreeding progression of Banna minipig inbred line. Animal Genetics.

[bib1.bib14] Itman C, Wong C, Whiley PA, Fernando D, Loveland KL (2011). TGF
β
 superfamily signalling regulators are differentially expressed in the developing and adult mouse testis. Spermatogenesis.

[bib1.bib15] Klein C, Rutllant J, Troedsson MH (2011). Expression stability of putative reference genes in equine endometrial, testicular, and conceptus tissues. BMC Research Notes.

[bib1.bib16] Li HJ, Yu N, Zhang XY, Jin W, Li HZ (2010). Spermatozoal protein profiles in male infertility with asthenozoospermia. Chinese Medical Journal.

[bib1.bib17] Li J, Hu WX, Luo SQ, Xiong DH, Sun S, Wang YP, Bu XF, Liu J, Hu J (2019). Promoter methylation induced epigenetic silencing of DAZAP2, a downstream effector of p38/MAPK pathway, in multiple myeloma cells. Cellular Signalling.

[bib1.bib18] Liu Y, Liang G, Wang H, Liu Z (2019). MicroRNA-129-5p suppresses proliferation, migration and invasion of retinoblastoma cells through PI3K/AKT signaling pathway by targeting PAX6. Pathology-Research and Practice.

[bib1.bib19] Liu Z, Zhang X, Huang L, Huo H, Wang P, Li W, Dai H, Yang F, Fu G, Zhao G, Sun YH (2023). Long-and short-read RNA sequencing from five reproductive organs of boar. Scientific Data.

[bib1.bib20] Lukas J, Mazna P, Valenta T, Doubravska L, Pospichalova V, Vojtechova M, Fafilek B, Ivanek R, Plachy J, Novak J, Korinek V (2009). Dazap2 modulates transcription driven by the Wnt effector TCF-4. Nucleic Acids Research.

[bib1.bib21] Mitchell SF, Parker R (2014). Principles and properties of eukaryotic mRNPs. Molecular Cell.

[bib1.bib22] Montjean D, De La Grange P, Gentien D, Rapinat A, Belloc S, Cohen-Bacrie P, Menezo Y, Benkhalifa M (2012). Sperm transcriptome profiling in oligozoospermia. J Assist Reprod Genet.

[bib1.bib23] Nilsen TW, Graveley BR (2010). Expansion of the eukaryotic proteome by alternative splicing. Nature.

[bib1.bib24] Niu Z, Goodyear SM, Rao S, Wu X, Tobias JW, Avarbock MR, Brinster RL (2011). MicroRNA-21 regulates the self-renewal of mouse spermatogonial stem cells. Proc Natl Acad Sci U S A.

[bib1.bib25] Nosková A, Wurmser C, Crysnanto D, Sironen A, Uimari P, Fries R, Andersson M, Pausch H (2020). Deletion of porcine BOLL is associated with defective acrosomes and subfertility in Yorkshire boars. Animal Genetics.

[bib1.bib26] Poliseno L, Salmena L, Zhang J, Carver B, Haveman WJ, Pandolfi PP (2010). A coding-independent function of gene and pseudogene mRNAs regulates tumour biology. Nature.

[bib1.bib27] Rao X, Huang X, Zhou Z, Lin X (2013). An improvement of the 2 (– delta delta CT) method for quantitative real-time polymerase chain reaction data analysis. Biostat Bioinforma Biomath.

[bib1.bib28] Rong SHEN, Wei REN, Li-jun TANG, Da-ren TAN, Wei-xin HU (2007). Molecular features and expression of DAZAP2 in human multiple myeloma. Chinese Medical Journal.

[bib1.bib29] Saitou M, Yamaji M (2012). Primordial germ cells in mice. Cold Spring Harb Perspect Biol.

[bib1.bib30] Shi Y, Luo S, Peng J, Huang C, Tan D, Hu W (2004). The structure, expression, and function prediction of DAZAP2, a down-regulated gene in multiple myeloma. Genomics Proteomics Bioinformatics.

[bib1.bib31] Tian H, Cao YX, Zhang XS, Liao WP, Yi YH, Lian J, Liu L, Huang HL, Liu WJ, Yin MM, Liang M (2013). The targeting and functions of miRNA-383 are mediated by FMRP during spermatogenesis. Cell Death and Disease.

[bib1.bib32] Tsui S, Dai T, Roettger S, Schempp W, Salido EC, Yen PH (2000). Identification of two novel proteins that interact with germ-cell-specific RNA-binding proteins DAZ and DAZL1. Genomics.

[bib1.bib33] Wang L, Sun J, Han J, Ma Z, Pan M, Du Z (2021). MiR-181a promotes spermatogenesis by targeting the S6K1 pathway. International Journal of Stem Cells.

[bib1.bib34] Wang P, Zhang X, Huo H, Li W, Liu Z, Wang L, Li L, Sun YH, Huo J (2023). Transcriptomic analysis of testis and epididymis tissues from Banna mini-pig inbred line boars with single-molecule long-read sequencing. Biology of Reproduction.

[bib1.bib35] Wilson ML, Romano SN, Khatri N, Aharon D, Liu Y, Kaufman OH, Draper BW, Marlow FL (2024). Rbpms2 promotes female fate upstream of the nutrient sensing Gator2 complex component Mios. Nature Communications.

[bib1.bib36] Xie X, Li H, Pan J, Han X (2021). Knockdown of TRIM26 inhibits the proliferation, migration and invasion of bladder cancer cells through the Akt/GSK3
β
/
β
-catenin pathway. Chemico-Biological Interactions.

[bib1.bib37] Zeng C, He L, Peng W, Ding L, Tang K, Fang D, Zhang Y (2014). Selection of optimal reference genes for quantitative RT-PCR studies of boar spermatozoa cryopreservation. Cryobiology.

[bib1.bib38] Zhang LY, Liu M, Li X, Tang H (2013). miR-490-3p modulates cell growth and epithelial to mesenchymal transition of hepatocellular carcinoma cells by targeting endoplasmic reticulum-Golgi intermediate compartment protein 3 (ERGIC3). Journal of Biological Chemistry.

[bib1.bib39] Zhang X, Huo H, Fu G, Li C, Lin W, Dai H, Xi X, Zhai L, Yuan Q, Zhao G, Huo J (2024). Long-read and short-read RNA-seq reveal the transcriptional regulation characteristics of PICK1 in Baoshan pig testis. Animal Reproduction.

